# Latent Dirichlet allocation model for world trade analysis

**DOI:** 10.1371/journal.pone.0245393

**Published:** 2021-02-04

**Authors:** Diego Kozlowski, Viktoriya Semeshenko, Andrea Molinari

**Affiliations:** 1 DRIVEN, FSTM, University of Luxembourg, Esch Sur Alzette, Luxembourg; 2 Universidad de Buenos Aires, Facultad de Ciencias Económicas, Buenos Aires, Caba, Argentina; 3 CONICET-Universidad de Buenos Aires, Instituto Interdisciplinario de Economía Política de Buenos Aires, Buenos Aires, Caba, Argentina; Consejo Nacional de Investigaciones Cientificas y Tecnicas, ARGENTINA

## Abstract

International trade is one of the classic areas of study in economics. Its empirical analysis is a complex problem, given the amount of products, countries and years. Nowadays, given the availability of data, the tools used for the analysis can be complemented and enriched with new methodologies and techniques that go beyond the traditional approach. This new possibility opens a research gap, as new, data-driven, ways of understanding international trade, can help our understanding of the underlying phenomena. The present paper shows the application of the Latent Dirichlet allocation model, a well known technique in the area of Natural Language Processing, to search for latent dimensions in the product space of international trade, and their distribution across countries over time. We apply this technique to a dataset of countries’ exports of goods from 1962 to 2016. The results show that this technique can encode the main specialisation patterns of international trade. On the country-level analysis, the findings show the changes in the specialisation patterns of countries over time. As traditional international trade analysis demands expert knowledge on a multiplicity of indicators, the possibility of encoding multiple known phenomena under a unique indicator is a powerful complement for traditional tools, as it allows top-down data-driven studies.

## Introduction

The role that countries play in the global market is profoundly determined by their insertion into global value chains, and by the types of goods they produce for the global market [1–3].

Production systems, which were traditionally analysed as almost independent national systems, are now continuously connected on a global scale. Due to the increasingly complex and interconnected nature of global supply chain networks, a recent strand of research has applied network science methods to model global supply chain growth and subsequently analyse various topological features of these structures. Clearly, this depends on the dataset in use, as it defines the topology of the network.

In recent years, we have been witnessing a continuous growth of available data. This situation also poses a great challenge, namely, how to extract hidden relations, determine appropriate patterns, clusters, and trends to extract valuable conclusions from such large volumes of data [4].

Traditional analysis tools are incapable to handle such complexity alone because it requires time and effort to extract and analyse information. On the other hand, interdisciplinary sciences provide different techniques and tools to apply to the analysis of this volume of data. The application of network formalism in the field of socioeconomic science has experienced unprecedented growth in recent decades [5–8]. Moreover, there is a wide literature that studies international trade at the product level [9–12]. In particular, these connections can be analysed as a bipartite graph between countries and products [13–16], and the complexity of production can be explored in the product space [17–19]. The world trade network can also be examined using multiplex and multilayer networks [20–22].

In this paper, we adopt a different approach to extract interesting and significant patterns from bilateral trade data, using the Latent Dirichlet Allocation (LDA) modelling technique [23]. Topic models have emerged as an effective method for discovering useful structures in data. At the same time, LDA is a statistical approach used in topic modelling for discovering hidden topics in large corpora of text.

Recently, a growing number of researchers are beginning to integrate topic models into various datasets [24–28], not only for text corpus. To the best of our knowledge, our work is the first effort to adapt and apply this technique for countries’ exports.

We find very suitable an analogy between topic modelling in texts and trade. In our adaptation of LDA, a set of countries plays the role of text documents, products play the role of words, and components (i.e. latent dimensions within which these product groups) play the role of topics. Based on the model of Blei et al. [23], we suggest a generative process to detect these latent dimensions in the product space and build an alternative trade nomenclature directly from data. Then, using these latent dimensions, we analyse the participation of those components within countries’ export baskets.

Our main contributions and results can be summarised as follows: based on a well established methodology usually used in the field of Natural Language Processing, we develop a generative model to study the international trade flows. This model looks for automatic grouping of the products in latent components. We study these latent components, characterising each by type of production, complexity and its relation to a specific country over time. Then, we use the components to briefly characterise the role in global trade of different groups of countries. The results that emerge from our model are in line with the specialised economic and trade history literature.

This model allows to use a single framework, with a minimum number of decisions, to characterise the role of countries in global trade. The obtained results are fine-grained enough to find differences and similarities between countries within the same general exports pattern.

The Topic modelling approach provides a data-driven summary of trade datasets, allowing the exploration of countries’ exports patterns with ease, enabling quickly find the similarities and differences in the export patterns of countries and detect structural changes along time. It does not replace qualitative interpretation, but rather complements it by enabling a degree of automated classification before the interpretive stage. This is a proposal from exploratory data analysis for trade that goes beyond traditional summary statistics.

The paper is organised as follows. In the next section we describe the dataset in use, introduce the notations, and explain the methodology applied in the model. Next, we present the obtained results. Finally, we conclude and discuss the benefits and limitations of this approach.

## Methods and data

### Data

To apply the LDA technique, we used the United Nations Commodity Trade Statistics Database (COMTRADE) dataset of each country’s (four-digits) disaggregated exports from the Center for International Development at Harvard University (extracted on March, 2019). Such dataset contains trade data for around 250 countries and territories, and takes the raw trade data on goods from countries’ reported to the United Nations Statistical Division.

We used these data instead of the raw COMTRADE statistics because such data may contain some inconsistencies. To address this issue, the Center for International Development uses the Bustos-Yildirim Method to clean data and “account for inconsistent reporting practices and thereby generate estimates of trade flows between countries”. This method assumes that since these data are recorded both as exports and as imports, cross-referencing countries’ reported trade flows against each other can produce reliable estimations. It consists of first correcting bilateral import values and then comparing them to the reverse flows reported by the exporting partner (see https://atlas.cid.harvard.edu/about-data for more details). Imports are reported including freight and insurance costs, and exports as free on board. Their per-country estimated index of reliability for reporting trade flows measures the consistency of trade totals reported by all exporter and importer combinations over time. Finally, they generate their own trade values’ estimates using the data reported by countries together with such reliability index.

Bilateral trade flows are mainly recorded in two trade classification systems: Harmonised System (HS) and Standard International Trade Classification (SITC), and the data presents four dimensions: exporter, importer, product, and year. While both classifications are valid, there is a “time versus disaggregation” trade-off entangled in the decision of which dataset to use. SITC data has a longer time-series (1962-2016), but it covers fewer goods (i.e. at higher levels of aggregation, up to 4-digits, approximately 750 products). On the other hand, HS data, being a newer classification, offers a more contemporary and detailed classification of goods (i.e., disaggregated up to 6-digits, with approximately 5,000 goods), but with the downside of offering a shorter period (1995-2017).

We chose to work with SITC (Revision 2) in order to have a larger time series, having slightly more aggregated data (i.e. 4- instead of 6-digits) [29]. Moreover, we reckon that 750 products allow for enough (but not too much) granularity when labelling the components. For such dataset, we make an empirical search for the best number of latent dimensions.

### Methodology

In this section, we describe a probabilistic model used to study the trade flow data with the aim to generate an automatic grouping of the products.

This cannot be achieved using traditional clustering techniques in high dimensional space [30], due to the fact that a product can be used or consumed as an intermediate and/or final product at the same time, which means that groups can not be exclusive [31]. Therefore, the problem we are dealing with can be examined with *fuzzy* clustering.

At the same time, we need to deal with mitigating high-dimensional data issues through dimensionality reduction. This is possible due to the fact that we can exploit the similarities between the products. The dimension of the problem of grouping the products can be thought of as a $R^{N*P*Y}$ space. That is, the interaction of *N* countries, *P* products and *Y* years.

We find it appropriate to use LDA to group products. While Blei et al. [23] look for a latent dimension of *k* *topics*, embedded in a highly dimensional dictionary distributed over the texts that compose the corpus, here we are looking for latent dimensions of *k* *components*, embedded in a highly dimensional classification of products distributed along the countries over the years.

We use the following terms to define our probabilistic topic model:

- ***product*** is a *basic discrete unit of analysis*, defined as an item in a classification (SITC). We represent products using unit-basis vectors, where the superscript *i* stands for the *i*^*th*^ product in the classification and the *i*^*th*^ element in the vector. The *V*^*th*^ product of the classification is the vector *w*, such that *w*^*v*^ = 1 and *w*^*u*^ = 0, *u* ≠ *v*.
- ***country-year*** is a sequence of **N** products, defined as *W* = (*w*_1_, *w*_2_, …, *w*_*N*_).
- **corpus** is the collection of **M** country-years, defined as *D* = (*d*_1_, *d*_2_, …, *d*_*M*_).
- ***component*** is a latent dimension on the corpus, defined as *K*.

The objective behind the classification of the products is twofold: on the one hand, look for a distribution of components over each country-year; on the other, analyse the distribution of the products within each of the components.

#### Generative process

In the original model proposed by Blei et al. [23], the words are supposed to be random realisations of chained distributions, ignoring the order in which the words appear in the document. Even when we know that the real data generating process is far from what our model proposes, this inference process can still provide useful insight on the latent dimensions we are looking for. The basic idea of the generative process is that, given the amount of dollars exported by a country in a specific year, the assignment of the product that will be exported comes from a random mixture over latent components, where each component is characterised by a distribution over products. The sequence of data generation can be described as follows:

- For each country-year in the corpus, we assume that exports come from a following two-stage process:
  - choose randomly a distribution for the components,
  - for every dollar exported:
    - choose randomly the component to which it belongs, and
    - choose randomly a product from the distribution corresponding to that component.

The data generating process can be formalised as follows:

1. For every component *k*_*i*_ ∈ {1, 2, …*k*}
  - Generate a distribution over the products *β*_*k*_ ∼ *Dir*(*η*), where $\eta \in R_{>0}$ is fixed
2. For each country-year *d* ∈ {1, 2, …*D*}
  - Generate a vector of component proportions *θ*_*d*_ ∼ *Dir*(*α*), where $\alpha \in R_{>0}$ is fixed
  - For every exported dollar:
    (a) generate an allocation of the component *z*_*dn*_ ∼ *Mult*(*θ*_*d*_)
    (b) assign the product *w*_*dn*_ ∼ *Mult*(*β*_*zn*_)

where both *η* = 1/*k* and *α* = 1/*k*.

A Dirichlet process is a family of stochastic processes where the realisations are themselves probability distributions. It is often used in Bayesian inference to describe the prior knowledge about the distribution of random variables—how likely it is that the random variables are distributed according to one or another particular distribution.

The parameters defining the Dirichlet distribution (here, *η* and *α*) determine the degree of concentration of the resulting distributions. For a *Dir*(*α*) distribution, *α* defines the degree of symmetry of the multinomial distributions that the process generates. With values much smaller than 1, the resulting distributions will be highly concentrated on some elements, while values much larger than 1 would generate very uniform distributions. In terms of our problem, *α* controls the mixture of components for any given country, and parameter *η* controls the distribution of products per component. A very small *α* will generate that each country has few characteristic components, while a very small *η* will generate a very asymmetric distribution over the products, and therefore there will be a few essential products, and the rest with almost null probability.

## Results

In this section we present the results of applying the aforementioned method to trade data. We confine our analysis to the 1962-2016 period, for 250 countries and products (goods, not services) reported in the mentioned dataset (in SITC, 4-digits [29]). In other words, we work within an order of magnitude similar to that of a regular dataset in a traditional (text corpus) Topic Modelling problem. As mentioned before, to prioritise a longer time series, we decided to use the SITC (as opposed to the HS) nomenclature.

In the following sections, in the first place we explain the setup of the model parameters, then the results are discussed in two stages. We first walk the reader through the decisions of the number of components and the labelling process adopted, to then analyse the evolution of exports in a selection of countries.

### Model setup

Here, we explain the (granularity versus economic interpretation) trade-off faced when using trade data with LDA. We describe the process of finding the best suitable number of components (*k*) for our problem and the labelling of each component, to finally reflect about our findings for the chosen *k*.

As mentioned, the hyperparameter *k* stands for the total number of components and plays a fundamental role in the model. Fewer components (i.e., small *k*) will tend to reflect broader concepts. On the other hand, a *k* larger than the cardinality of the latent space (i.e., the implicit space for the grouped products is smaller than the number of proposed components), can generate repeated or over specific components. In other words, in our case this issue poses a trade-off between granularity and well-defined (i.e. easily “taggable”) components.

We ran the model for different values of *k*: 2, 4, 6, 8, 10, 20, 30, 40, 50, 100, 200. The first result, observed for any value of *k*, is that the components which group the best are those containing: petroleum and derivatives, electronics, machinery, and textiles. As mentioned above, the hyperparameter *k* defines the components’ specificity. However, those phenomena worth exploring (and for economic interpretation) can be found at different levels of granularity. Hence, the first problem found when analysing the different exercises is to define a suitable granularity for the components. For relatively low values of *k* (i.e. up to *k* = 10) the petroleum component always stands out. Conversely, for *k* = 20 we also find other sectors (e.g., electronic products, textiles, etc) in some components, while others hold a mixture of products that is harder to rationalise as a latent dimension. For values of *k* between 20 and 50, the resulting composition for each component is rather stable, resulting in a good balance between more easily interpretable (i.e. taggable) components, together with an interesting level of granularity. For values of *k* higher than 50 components tend to repeat themselves.

The next step consisted of choosing a value for *k*, considering the granularity versus taggability trade-off. There is no single way for searching an optimum value for *k*, and although the literature within the text analysis domain has contributed with some proposals, the setup for *k* comes from a substantive search where the topics (or components) found are closer to the object under study [32, 33].

To define the most suitable value for *k* and to label the components, we developed a dynamic dashboard (see https://ldaglobaltrade.uni.lu/dashboard/) with the distribution of products over components and their cumulative share. We also include Lall’s classification [10], which divides traded goods by degree of processing (primary products, resourced based manufactures, and non-resourced based manufactures) and, for industrial products, complexity (low, medium or high). For each component, we project the distribution as a weighted average of Lall’s groups, using the share of each product in the component. Given that not all SITC products are classified by Lall, some of them are grouped as “Unclassified”.

The characterisation of the model for different *k*, and the posterior labelling of components is a process that includes the following steps:

1. We first decided the quantity of products to analyse. This was done on the basis of the cumulative share distribution of the top products (up to 10 with the highest share). The more concentrated (following the cumulative probability), the less products are needed for a good characterisation.
2. We then defined a concept that generalises products with the highest share. For example, for *k* = 30, in component 1 the first four products are *coal*, *iron*, *gold* and *aluminium*, which can then be labelled as *Minerals*.
3. For components where the top 10 products have a cumulative share of less than 30%, we looked at the overall distribution of the component in Lall’s groups:
  - If the distribution is skewed, this means that the component is still well defined, but includes multiple products of the same type (see for example, component 4 for *k* = 30),
  - If the distribution is uniform, this means that the component is ill defined (see for example, component 2 for *k* = 2).
4. After studying all the components of different models (i.e. different values of *k*), we selected the model that satisfies the following criteria:
  - It is feasible to label most of the *k* components;
  - Components do not repeat among themselves;
  - The distribution of components gives a high cumulative share (more than 30%) for the first 10 products in the majority of the components.

As a result, we found that *k* = 30 gives the best trade-off between having enough (economically meaningful) granularity and a relatively low components’ repetition. Moreover, the fact that models with *k* = 20, 40 do not derive in too different findings indicate that the model is robust to variations of *k* near the selected value 30. Henceforward, this model is used in our exploratory analysis.

In addition, as an example, S1 Appendix shows the step-by-step labelling exercise and some possible economic interpretation of results for *k* = 2.

We also tested for different values of *η*. As we want our components to have an asymmetric distribution, in order to facilitate their labelling, we ran the model with small values of *η*. Specifically, we tested the model for *η* = 1/30, 1/60, 1/90, 1/120. Components’ composition did not show substantive changes for different values of *η*, indicating that the model is robust to variations in the priors. For this reason, and given that the default value $\alpha =\frac{1}{k}$ gives good results in terms of countries specialisation, we keep the default values of $\alpha =\eta =\frac{1}{k}$ for various runs for different values of *k*.

### Analysis of components

Frequently when topic modelling is applied to text data, the labelling process may result difficult due to the potential lack of generalisation criteria. This also occurs in the case of export data, since the subjective search for a comprehensive concept of products traded among countries can turn to be a more complex task than searching for a general concept over a group of words. On the upside, polysemy, a frequent problem found in texts, does not exist in trade data, where all signifiers (classification indexes) refer to a single and unambiguous meaning. However, new problems arise, e.g. deciding upon the trade nomenclature or the data disaggregation level (which could be associated with choosing the language of the corpus in text analysis). In our model, we first observed that the usual practice of looking at the first ten elements of the distribution is not sufficient to find a general label for each component, and for this reason we complement the analysis with the dashboard, that includes the most relevant product, their shares and cumulative shares, and the projection of the component into Lall’s categories in case they are needed.

Table 1 shows the labels for our model (*k* = 30), with a general description of components, except when that was not possible (e.g., component 19), together with a ‘subgroup’ that allows for a more detailed product specification and, in the case of industrial products, the level of technological complexity (according to Lall [10]). Finally, the last column displays the country for which each component has the highest share (taking an average over the whole period).

**Table 1:** Latent components. Groups, industrial complexity and representative country. k = 30.

Group	Comp	Subgroup	Complexity	Country
Industry	2	Textiles, engineering, others	Low and medium	San Marino
Industry	3	Vehicles and parts	Medium	Belgium
Industry	4	Footwear, clothing and toys.	Low	Macao
Industry	5	Non-digital electronics, record tapes, telephone lines, photographic paper	High (up to 70’)	Czechoslovakia
Industry	6	Vehicles, boats, machinery and parts	Medium and high	Japan
Industry	10	Cars and electronics	Medium and high	Mexico
Industry	11	Cars, parts and other machinery	Medium	Germany
Industry	14	Lubricating petroleum oils and preparations and other chemicals	-	Curaçao
Industry	21	Medicaments, medical appliances and chemicals.	High	Irlanda
Industry	23	Processors, microcircuits, toys and shoes.	High and low	China
Industry	27	Electronic microcircuits and other machinery parts.	High	Philippines
Industry	30	Vehicles, parts and medicines	Medium and high	United Kingdom
Industry + Agro.	16	Aircraft, auto parts, soya and corn	Medium and high	USA
Industry + Agro.	17	Vehicles, parts, wood and derivatives	Medium	Finland
Industry + Agro.	18	Primary Products and textiles	Low	Christmas Island
Industry + Agro.	24	Boats, meat, fish, dairy	Medium	Iceland
Industry + Agro.	26	Aircrafts, vehicles, perfumery, wine.	High	France
Industry + Agro.	28	Rice, cotton, textiles, gum, etc.	Low	Pakistan
Industry + Agro.	29	machinery, flowers, cheeses.	High	Netherlands
Minerals	1	Coal, iron and other primary products (wheat, meet, wool)	-	Australia
Minerals	8	Copper	-	Chile
Minerals	15	Diamonds	-	Botswana
Oil	7	Petroleum gases	-	Turkmenistan
Oil	12	Crude petroleum	-	South Sudan
Fuels	20	Fuel oil, gasoil, etc.	-	Yemen
Oil + Agro	22	Hydrocarbons, palm oil, cocoa, etc.	-	Ghana
Minerals + Agro.	25	Soya and derivatives, Iron	-	Paraguay
Agricultural	9	Coffee, bananas, other food and primary products	-	Reunion
-	13	Gold, watches, jewelry	-	Switzerland
-	19	Unclassified Special transactions	-	St. Maarten Island

It is interesting to particularly highlight component 5, albeit (as mentioned below) it is not defined for a few products, given its high technological complexity (recording tapes, telephone lines, or photographic paper) at the beginning of the series (during the ’60s), but which later fell into disuse. In this sense, it is unsurprising that Czechoslovakia would be the most characteristic country of this component, given that, due to the country’s dissolution in 1992, its time series is shorter than the rest. In S2 Appendix shows the average share by decade of those countries with the highest proportion of this component.

The following is a summary of some of the regularities identified in the results for our LDA model, by looking at each component and with the aim of understanding what could such results reflect in terms of product composition (or exporting basket). To do so, we analyse the granularity and homogeneity of each component and confront its products with the export basket of the main country identified.

In 23 out of the 30 components, the first ten 4-digits products explain a cumulative share over 30%, i.e., can be studied in more details looking at a small number of products. This includes one component (19) which groups, with a 96% share, unclassified commodities (“Special transactions, commodities not classified according to class”).

In general terms, our LDA model seems to capture countries with a strong export basket concentration, either at the beginning´, or mostly, at the end of the period. In other words, those countries that have a high concentration of a certain product in their export basket tend to be the main actors in the component that concentrates such product. Moreover, the 1962-2016 time series allows us to find important structural changes in the countries’ export basket. A brief characterisation of what our LDA model may be capturing over time can be divided into five groups.

On the one hand, nine (out of the mentioned 23) components show a main country with significant export increases. First, Turkmenistan (the main country in component 7) “Petroleum gases, nes, in gaseous state” exports rose from 0.1% (in 1995) to 73% (in 2016), while Philippines’ export share of “Electronic microcircuits” (component 27) grew from 0.01% (in 1971) to become its first exporting good (with 27.2% over total goods’ exports in 2016). Other impressive increases are shown by Ireland (component 21, with “Medicaments (including veterinary medicaments)” going from 0.1% to 15.2%), and China, with exports of “Television, radio-broadcasting; transmitters, etc” (component 23) rising from null to 6% (becoming its main export product, even including services, in 2016). Furthermore, Australia (component 1) saw a rise in “other coal, not agglomerated” exports from 1% (in 1962) to 14.1% (2016), while looking at component 6, Japanese exports of “Passenger motor vehicles (excluding buses)” grew from 0.6% (1962) to 13.9% (2016). In component 10, Mexico’s exports of “Passenger motor vehicles (excluding buses)” grew from 0% to 8% over the period. Moreover, french export shares of “Aircraft of an unladen weight exceeding 15000 kg” (main product in component 26), went from 0.3% to 8.6%. Moreover, in component 25, the second and third products are significant in terms of Paraguayan exports and show important rises: “Oilcake and other residues (except dregs)” increased from 2% to 12.3% and sales of “Soya beans” from 0.3% in 1963 to 23% (becoming the country’s main exporting product, even including services, in 2016). Moreover, British exports of “Passenger motor vehicles (excluding buses)” remained practically stable (5.2 to 5.3%), although the following relevant products in component 30 (“Parts, nes of the aircraft of heading 792” and “Medicaments (including veterinary medicaments)”) saw significant increases (from 0.3% to 3.6%, and from 0.6% to 5.3%, respectively).

The second group of (three) components shows significant falls over the period. In component 8, Chilean exports of “Copper and copper alloys, refined or not, unwrought” fell from 30.3% in 1962 to 22.6% in 2016, while “Copper ore and concentrates; copper matte; cement copper” exports decreased from 33.1% to 19.1%. Also, Finish (component 17) “Wood of coniferous species, sawn, planed, tongued, grooved, etc” exports fell from 21.4% (1962) to 2.7% (2016), while Pakistan (component 28) saw a shrinking share of “Raw cotton, excluding linters, not carded or combed” exports, from 9.8% to 0.2%.

The third group is formed by two components that show relatively constant trade over the period. In component 4, Macao experienced stable “Footwear” exports (from 4.1% in 1962 to 3.9% in 2016), and hence its emergence can probably be explained by its significant share in service exports (with tourism taking 88.8%). On the other hand, Germany (in component 11) exported 8% (in 1962) and 11.2% (in 2016) in “Passenger motor vehicles (excluding buses)”, although its preponderance can be due to the fact that it is the main world exporter of this good.

The fourth group shows another singularity of this LDA trade data application: in some (four) components it singles out countries with a short time series due to their shorter data history, as mentioned to explain Czechoslovakia in component 5. While Reunion data ranges over the 1962-1995 period and it mainly exports “Sugars, beet and cane, raw, solid” (the third main product from component 9, with a 4% probability), with its exports basket shows an important concentration of this product (albeit falling from 83.6% to 66.2%). South Sudan (the main country in component 12) exported 98.7% in “Crude petroleum and oils obtained from bituminous materials” in 2016, but it only presents data from 2012, while Kuwait was the main exporting country of this product in 1962 (albeit falling from 17.7% to 7.5% in 2016) and Saudi Arabia in 2016 (rising from 10.5% in 1962 to 18.2%). Moreover, Curaçao (component 14) presents data only for 2011-2016 and Botswana (component 15) from 2000 (with 64.3% probability in “Diamonds (non-industrial), not mounted or set” exports and rising to 88.3% in 2016), albeit the country only exported 1.4% of that product globally in 2000 (although that share grew to 4.7% in 2016).

The fifth group is composed by only one component (22) that does not show a particular regularity that can explain the representative country (Ghana): its main product (“Petroleum gases and other gaseous hydrocarbons, nes, liquefied”, with a 38% probability) is currently mainly exported by Qatar, rising from 0.2% (in 1975) to 22.4% (in 2016).

Finally, another interesting fact derived from our LDA model is that there is one product (“Passenger motor vehicles (excluding buses)”) captured as the main one in six of the 30 components (3, 6, 10, 11, 17 and 30). This seems to reflect the different exports specialisation in the main country for each component (respectively, Belgium, Japan, Mexico, Germany, Finland, and UK). As previously mentioned, Germany (component 11) has been the main exporter of this product over the whole period (albeit with a falling share from 37.6% to 22.1% over total exports), while the Japanese share (component 6) grew from 1.9% to 13.5%, those from UK and Belgium fell (from 19.6% to 5.9%, component 30; and from 4.7% to 3.8%, component 6; respectively), Mexico’s rose (from 0% to 4.7%; component 10), and Finland’s was the lowest (from 0.1% a 1.8%; component 17).

### Analysis of countries

Having labelled the components, in this section, we analyse each country’s export basket composition over the period under study (1962-2016). Our country-year unit of analysis allows us to compare the evolution in components’ distribution within each country.

**Fig 1:**
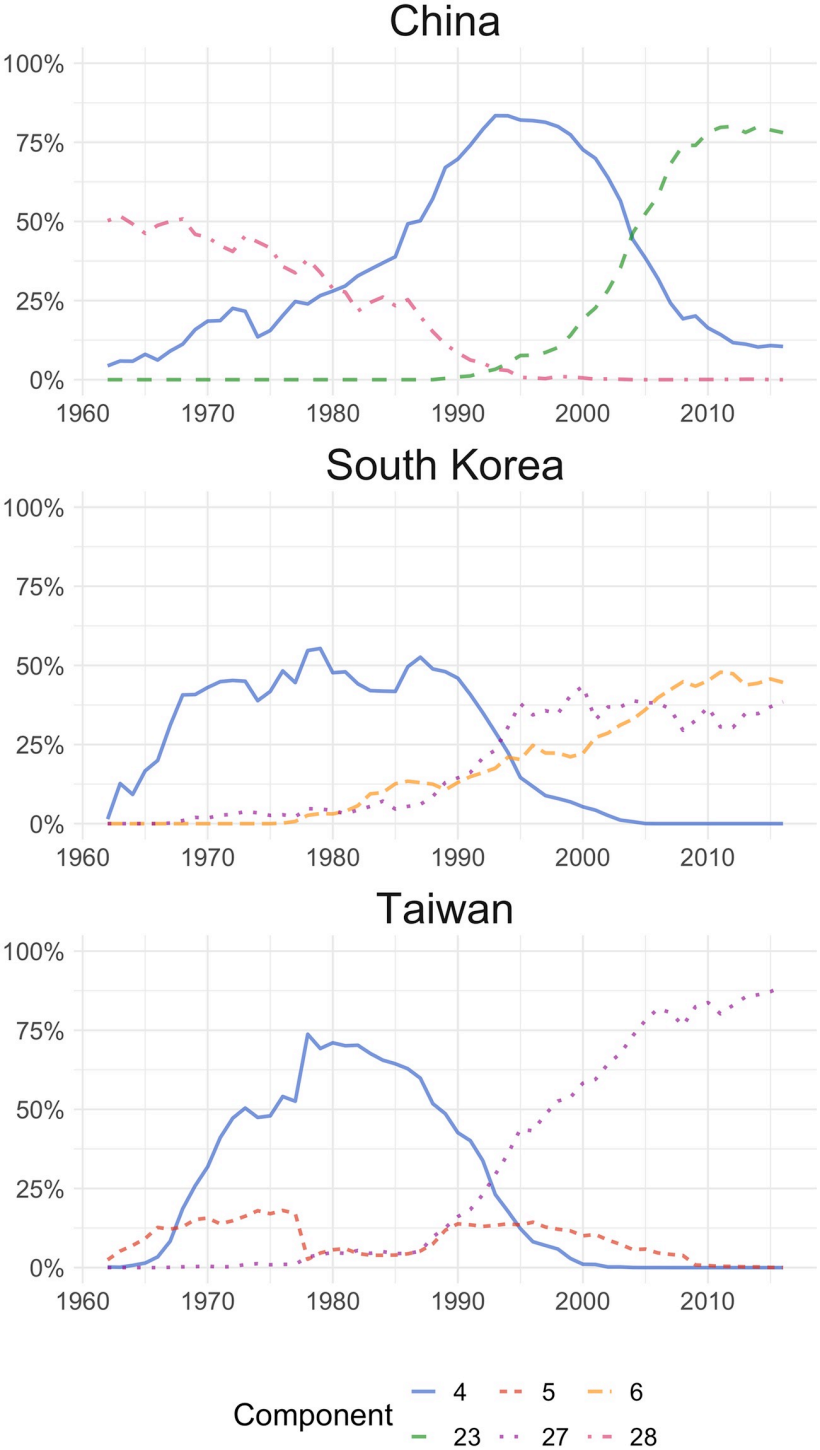
LDA outputs for China, South Korea and Taiwan. Distribution of the top three components by country. 4: Footwear, clothing and toys; 5: Non-digital electronics; 6: Vehicles, boats, machinery and parts; 23: Processors, microcircuits, toys and shoes; 27: Electronic microcircuits and machinery parts. 28: Rice, cotton, textiles.

Fig 1 shows the export structure of components in China, South Korea, and Taiwan. In the case of China, at the beginning of the ‘60s, the most relevant component (28) suggests an exports basket of rice, cotton, tea, and some textile products. This component shows a downward trend, while clothing, toys, etc. (component 4) becomes the most important over the period 1980-2003. However, from 1993, component 4 starts falling, with a simultaneous rise in component 23 (televisions, computers, microcircuits, and transistors), which towards the last years of the period analysed constitutes approximately 80% of the country’s exports. This change in Chinese export basket reflects three stages of increasing complexity of the country’s manufacturing industry, starting from a basically agricultural (or low tech) economy which, after a period of low-complexity industrialisation, becomes one of the world’s leading exporters of highly complex products [34, 35]. A similar behaviour is shown by South Korean and Taiwanese exports, where over the 60’s component 28 (rice, cotton, etc.) had more weight over total exports, losing importance by the mid 60’s and 90’s to be replaced by components 4 and 27 (respectively). It is worth mentioning that component 28 has also shown a significant and decreasing weight at the beginning of the Hong Kong series, suggesting that this country could have gone through a similar process as the other Asian countries described, albeit earlier. In the case of South Korea, along with component 27, component 6 (engines, ship, and electrical machinery) also becomes more relevant during the same time frame.

**Fig 2:**
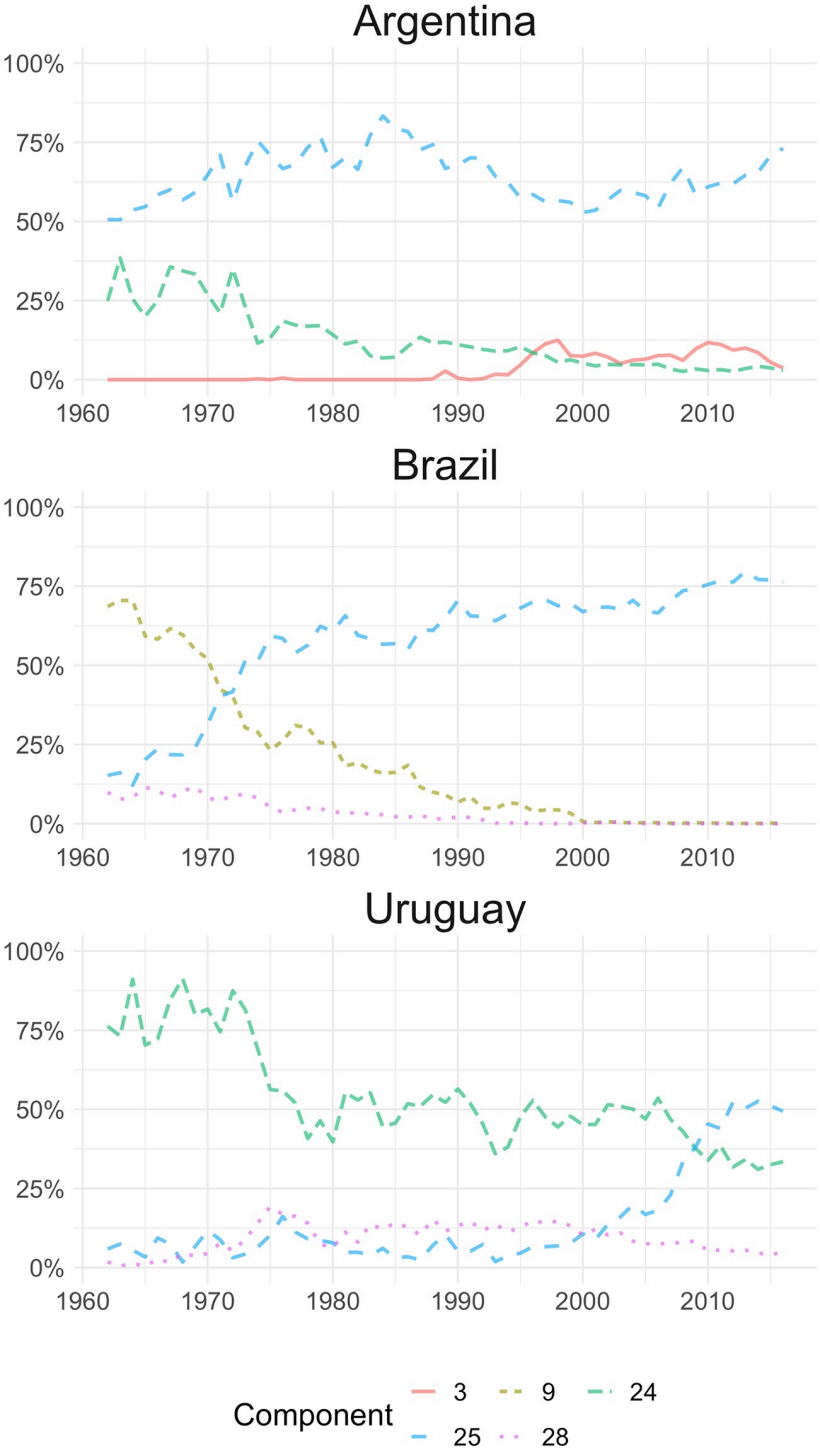
LDA outputs for Argentina, Brazil and Uruguay. Distribution of the top three components by country. 3: Vehicles and parts; 9: Coffee, bananas, other food and primary products; 24: Boats, meat, fish, dairy; 25: Soya and derivatives, Iron; 28: Rice, cotton, textiles.

Furthermore, Fig 2 shows the evolution of the main components in Argentina, Brazil, and Uruguay. In the case of Argentina, component 25 (primary products such as soybean and iron) is predominant over the whole period. At the beginning of the series, component 24 (livestock, fishing, and dairy products) is also relevant, but its weight decreases over time. Component 3 (automobiles) increase its share from the 90’s, which may follow the preferential trade policy for that sector since the creation of the Southern Common Market (MERCOSUR) [36]. In the case of Brazilian exports, the dominant component changes from 9 (coffee, bananas) to the mentioned component 25, although in this case, it is possible that iron exports are ahead of soybean [35]. In the case of Uruguay, unlike its two MERCOSUR partners, the series starts with a predominance of component 24 (livestock, fishing, and dairy), but since the mid-90’s the country’s exports lose importance to components 25 (soybean and iron), which from 2009 becomes the main component [37]. Moreover, component 28 (which includes textiles) keeps its share of the export basket along the series, unlike the Brazilian case, probably due to the tradition of that industry in the country [38].

**Fig 3:**
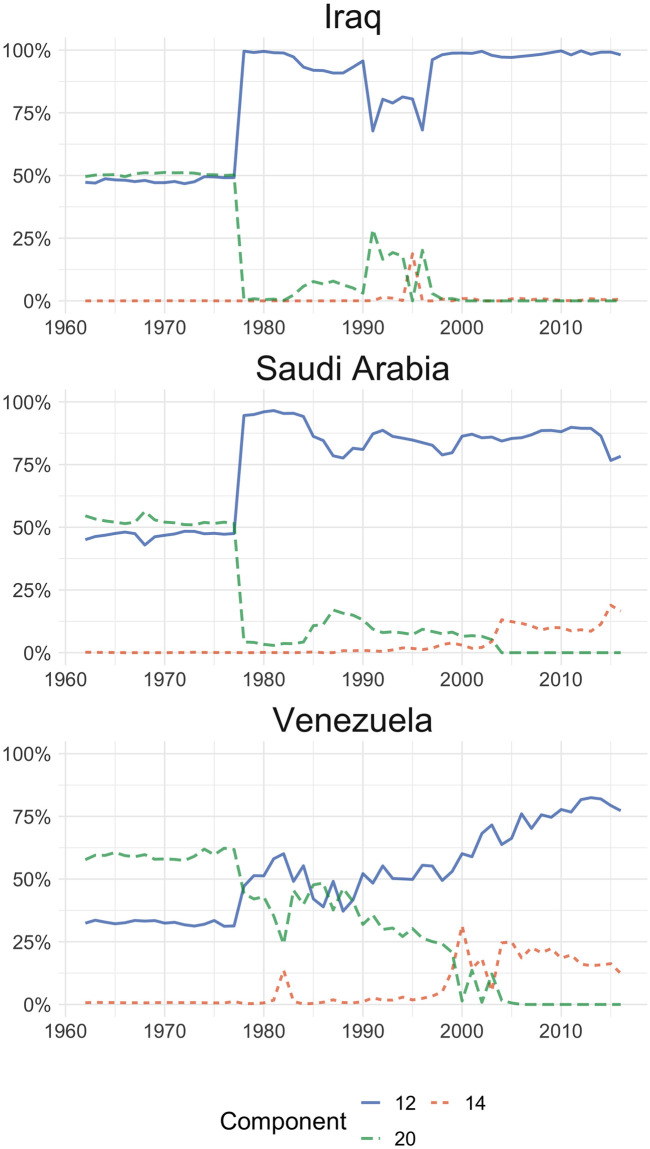
LDA outputs for Iraq, Saudi Arabia and Venezuela. Distribution of the top three components by country. 12: Crude petroleum; 14: Lubricating petroleum oils and preparations; 20: Fuel oil, gas-oil.

Another finding worth mentioning is the components’ distribution for three countries (Iraq, Saudi Arabia and Venezuela) members of the Organisation of Petroleum Exporting Countries (OPEC), shown in Fig 3. As expected, the exports baskets of these countries show a strong concentration in oil and oil derivatives. Prior to the 1979 oil crisis [39], exports were symmetrically divided between components 12 (crude petroleum) and 20 (fuels), but after this episode (with the rise in the price of a barrel of crude oil) the share of crude petroleum increased sharply and has remained so until the end of the period analysed. The case of Venezuela is particular, given that its fuel exports prior to the 1979 crisis had a greater weight than crude oil, and although this trend reverted after that year, during the 80’s and 90’s, component 20 continued to show an important share, although crude petroleum has been gaining relevance. Also, both Saudi Arabia and Venezuela show a relatively important share of lubricating oils and preparations exports (component 14), perhaps reflecting some value added to the mentioned prevailing commodity. In S2 Appendix we show the behaviour of mineral exporting countries.

Finally, it is worth mentioning some other findings in a previous work (see [40]). First, there is a particular national differentiation among EU countries’ export baskets, with a concentration in a single component that varies among countries, while most Asian exports tend to show much more homogeneous export baskets. Finally, our LDA model captures the export specialisation in electronic products in the United States, moving from analogue to digital technologies over the period of study, together with the *Maquila* phenomenon in Mexico (see Figures in S2 Appendix).

## Discussion

The present work proposes the use of a technique widely explored in Natural Language Processing in the field of international trade. By shifting the data domain from text to each country’s export flow of each product, we managed to develop a typology of global trade based on a number of latent components. This allowed us to do two things. On the one hand, we built an automatic classification of products based on data. On the other, we were able to study different trends in countries’ exports based on those components. Our findings are mostly in line with the specialised literature for each country or region, showing that this particular methodology is able to grasp an insight of the position of countries’s exports in global trade, making use of a single type of metric. International trade flows are a complex phenomenon which involves multiple countries, years and products. In order to understand specialisation patterns, expert knowledge and a multiplicity of indicators are needed for each country. Our model allows to have an overview of each country’ specialisation pattern using a single metric and with a minimum number of decisions (the number of topics). Given that all countries are described using a unique set of metrics, the latent components, it turns out to be an interesting complement to the traditional analysis, in order to develop a top-down data driven analysis. On the country level, the model results are fine-grained enough to not only characterise the general role of a country in global trade, but to differentiate it from others in that same typology. For example, in the case of Asia (see Fig 1) not only we can see how they move from low to high complexity products, it is also possible to distinguish the time-frame of the switch. Even in the case of countries with a really specific specialisation pattern, like copper exporters (see S2 Appendix), we can distinguish how Peruvian and Zambian exports baskets resemble more, also on those less important components, than the former with respect to Chile.

Nevertheless, one of the limitations of the proposed methodology is in its dependence of the data inputs. Decisions made with respect of the curation of the dataset can potentially affect all the results. If the dataset used starts in the beginning of the 20^th^ century, the resultant components would be very different to the ones presented in this article due to the larger set of technologies involved, and the selected number of components would probably increase. On the other hand, if a country is restricted to a subset of the years considered, it will have an overall closer relation with components specialised in technologies of that time-frame, like in the case of Czechoslovakia. Even when each country-year weights the same in the optimisation of the model (i.e., we are not considering the weight of total exports of each country-year on the cost function), countries with larger exports tend to show smoother results, as shown by the case of China. This is due to the fact that the higher exports volumes make it difficult for a specific product to drastically change its proportion in the country´s total exports from one year to another. Further, small countries are more prone to sudden changes in the proportion of components because a small change in the nominal value of their exports of any specific product imply a relatively larger proportion over the exports basket.

An also interesting phenomenon occurs with countries that have a highly concentrated export basket. For the OPEC countries we can see a dramatic change by the end of the 70’s. If we take the case of Iraq, for example, its exports basket goes from an equal distribution on components 20 and 12 to a 100% in the component 12, some years later. The distribution on the original SITC nomenclature shows that this country exported 61.68% in “Crude petroleum” and 36.5% “Petroleum products, refined” in 1977, and the next year this changed to 85.03% and 12.59% respectively. This implies an increase of more than 23% of the overall basket in a single product. Still, it is not a 50% change as showed by the proposed model. The explanation for this is that both latent components (12 and 20) include, with different proportions, crude and refined petroleum. The model infers that the refined petroleum exported from 1978 onwards comes from a different latent component than the one exported previously. It is possible to argue that if a country’s exports can be correctly described with only two products, then using a model like LDA is not necessary for studying its exports basket.

Benchmarking the results of the LDA model is a complicated task, as it is an unsupervised model. The best model should be the one that gives the most interpretable results, and that can be used for the more insightful analysis. To test our model, we tried three other approaches for the same task: finding the latent dimensions of international trade. First, we tried two other methods traditionally used for Topic Modelling in Natural Language Processing, namely, Latent Semantic Analysis [41] and Non-Negative Matrix Factorisation [42]. Then, we looked into the product space [18, 19] to achieve the same task as LDA by using clustering techniques [43]. The three techniques showed results that are in line with the ones found by LDA, but in a lower level of detail, hence making the interpretation of results an even harder task.

It is interesting to look at the feasibility of the model given the change in the domain of the problem. The very different nature of the data traditionally used in text mining and Topic Modelling, with respect to international trade data, raises the question whether the model can operate within the new domain. However, in terms of data structure, both problems have more similarities than what it seems. First, the traditional dimension of the problem is NxV (N observations, in the order of magnitude of thousands, V the vocabulary, also in the order of magnitude of thousands). In this case, the problem is approximately NxP, where the N observations are year-country pairs, with 250 countries and 54 years, and P products, which in SITC at 4 digits are approximately 750. In other words, we are working with an order of magnitude similar to that of a small dataset in a traditional Topic Modelling problem. Finally, an important change in both domains is the difference between the frequency of words in a text (tens or hundreds, depending on size of the document) and the dollars exported of a product by each country-year (millions or billions). This difference in principle should not affect the model, since what the model considers in its optimisation are the distributions between the different elements (word frequencies or exported values per product) and not the absolute values.

As future lines of work, as results are deeply connected to the input dataset, new data sources could provide different insights. For example, while our period seems long enough to reflect structural changes, economic historians could find an even longer time series more useful to describe some phenomena. Moreover, including services in the dataset could show different aspects of global trade that cannot be captured in an analysis only covering trade in goods. That said, data limitations would pose a trade-off, as this would imply either a lower product disaggregation or a shorter time series dataset. Other lines of work involve an exploration by country groups, to explore specialisation or complementarity among countries exports baskets, e.g., within a regional trade block (like the mentioned MERCOSUR).

As a final remark, this work is of an exploratory nature, and is our attempt to bring a new tool as a method for trade analysis, which helps to add different dimensions of analysis and visualisation. This method does not replace traditional metrics and empirical work on international trade, but rather complements traditional analysis and helps in the understanding of this field.

## Supporting information

S1 Appendix (PDF)

S2 Appendix (PDF)
