# Supplementary material for: Latent Dirichlet allocation model for world trade analysis
Source: PLoS One. 2021 Feb 4;16(2):e0245393. doi: 10.1371/journal.pone.0245393 (PMC7861422; doi:10.1371/journal.pone.0245393)
Supplement: S1 Appendix — (PDF) [file pone.0245393.s001.pdf]

S1 Appendix. Model with k=2. Figure S1_1 displays the mentioned interface for $k = 2$, showing each 4-digit SITC code and its product description, together with its individual and accumulated probabilities within the component. Further, Figure S1_1 shows that the distribution of the first component assigns a large weight to crude oil, followed by other petroleum products (e.g. diesel oil, propane gas, etc.). Hence, a plausible label for such component would be ”Petroleum and derivatives”. However, it is also worth noting that component 1 also holds other products such as coal and metals (e.g. iron, gold and copper). Figure S1_2 shows the distribution of the second component, which is more homogeneous than the first component, as the first product weights only 5 %, and the most outstanding products are passenger vehicles, electronic microcircuits, parts and accessories, etc. Hence, this component can be labelled to represent manufactured products in general.

Code	Description	Share	Cumulative Share
3330	Crude petroleum and oils obtained from bituminous materials	35%	35%
9310	Special transactions, commodity not classified according to class	6%	40%
3345	Lubricating petroleum oils, and preparations, nes	5%	46%
3414	Petroleum gases, nes, in gaseous state	4%	50%
3413	Petroleum gases and other gaseous hydrocarbons, nes, liquefied	4%	54%
3222	Other coal, not agglomerated	2%	56%
2815	Iron ore and concentrates, not agglomerated	2%	58%
9710	Gold, non-monetary (excluding gold ores and concentrates)	2%	60%
3344	Fuel oils, nes	1%	61%
6821	Copper and copper alloys, refined or not, unwrought	1%	63%

Showing 1 to 10 of 786 entries Previous 1 2 3 4 5 … 79 Next

Fig S1_1. Screenshot of the interface for component characterisation Highlighting of the proportion of the product in the component, and cumulative distribution. k=2. First component

Moreover, Figure S1_3 shows the components' distribution according to the mentioned classification developed by Lall [1]. The first component is essentially composed of primary products and manufactures that use primary products as inputs. On the other hand, component 2 presents a more uniform distribution, where medium and high technology manufactures (e.g. engineering and electronics) stand out.

However, it is worth noting that for $k = 2$, agricultural, livestock and forestry products cannot be singled out in one same component. That said, an interesting finding is that the division of the product space in only two groups allows the LDA model to find a first component mainly formed by petroleum (and its derivatives) products, while the other holds mostly manufactured products (SITC 5-8). In this sense, such model could allow understanding the classic corollary of comparative advantage models, where

Code	Description	Share	Cumulative Share
7810	Passenger motor vehicles (excluding buses)	5%	5%
7764	Electronic microcircuits	3%	8%
9310	Special transactions, commodity not classified according to class	3%	11%
7849	Other parts and accessories, for vehicles of headings 722, 781-783	2%	14%
5417	Medicaments (including veterinary medicaments)	2%	16%
3345	Lubricating petroleum oils, and preparations, nes	2%	18%
7599	Parts, nes of and accessories for machines of headings 7512 and 752	2%	20%
7649	Parts, nes of and accessories for apparatus falling in heading 76	1%	21%
7643	Television, radio-broadcasting; transmitters, etc	1%	22%
7721	Switches, relays, fuses, etc; switchboards and control panels, nes	1%	23%

Showing 1 to 10 of 786 entries   Previous 1 2 3 4 5 … 79 Next

Fig S1_2. Screenshot of the interface for component characterisation Highlighting of the proportion of the product in the component, and cumulative distribution. k=2. Second component

developed countries export manufactures (i.e. component 2) while developing countries specialise their trade in raw materials [2]. Some of the literature places a particular role to oil production (and exports) within an economy's structure [3, 4]. In this sense, with $k = 2$ oil-producing countries' exports seem to lead the LDA model in finding its optimum by building one of the two components with such products. However, this dichotomy should be taken with care in the case of petroleum. As Ross [3] states, the resource curse of oil producing countries may be biased upward in poorer countries when using their dependence on hydrocarbon exports and derive ”spurious associations between oil export dependence and a variety of economic and political maladies that are highly correlated with low incomes”. This is hence an arguable statement, as oil exports reflect an indirect measure of a country's non-oil economic size, although also the so-called ”Dutch Disease” in oil-exporting countries has often crowded out their agricultural and manufacturing exports due to the cited comparative advantage [3].

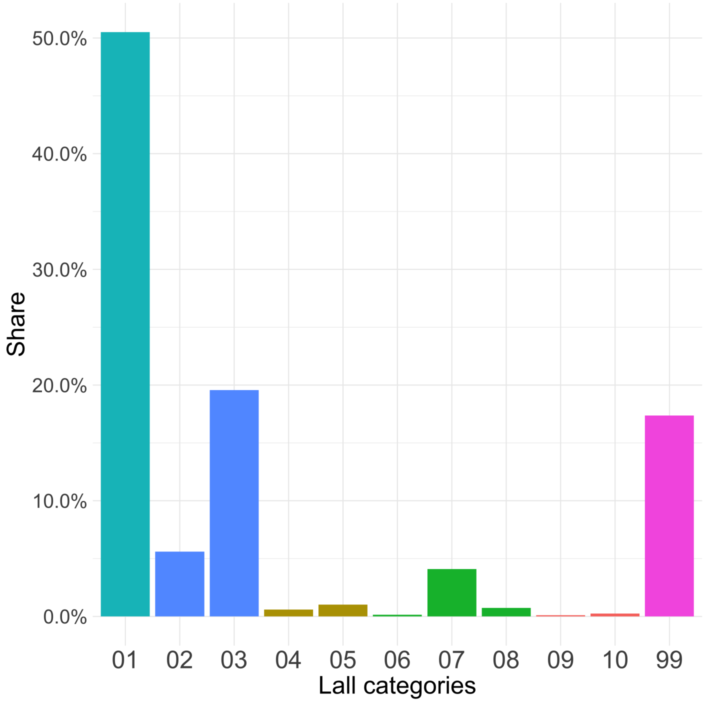

Fig S1_3. Distribution of components by Lall [1]. k=2. First Component. 01: primary products; 02: agro-resource-based manufactures; 03: non-agro-resource-based manufactures, 04: textile, garment and footwear (low-tech manufactures); 05: other low-tech manufactures; 06: automotive (medium-tech manufactures); 07: process (medium-tech manufactures); 08: engineering (medium-tech manufactures); 09: electronic and electrical (high-tech manufactures); 10: other high-tech manufactures; 99: unclassified products

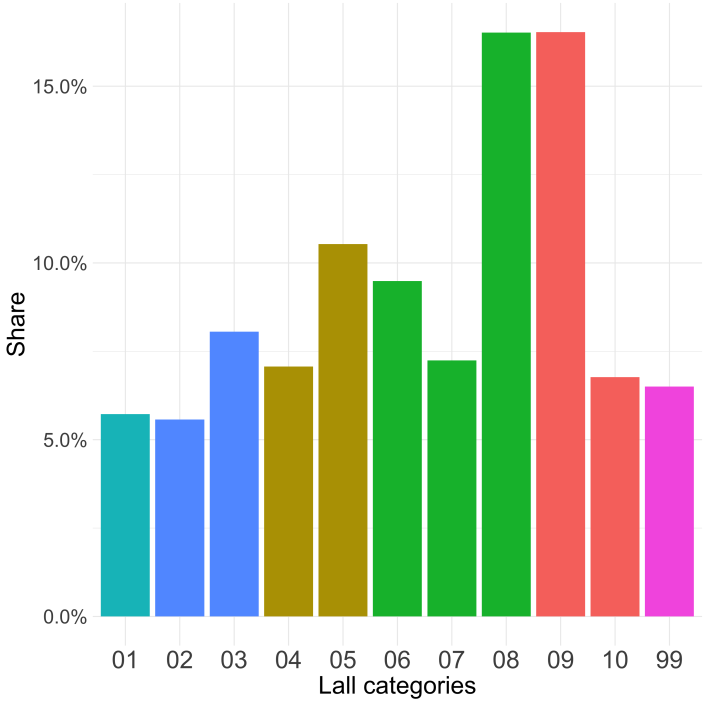

Fig S1_4. Distribution of components by Lall [1].k=2. Second Component. 01: primary products; 02: agro-resource-based manufactures; 03: non-agro-resource-based manufactures, 04: textile, garment and footwear (low-tech manufactures); 05: other low-tech manufactures; 06: automotive (medium-tech manufactures); 07: process (medium-tech manufactures); 08: engineering (medium-tech manufactures); 09: electronic and electrical (high-tech manufactures); 10: other high-tech manufactures; 99: unclassified products

References

1. Lall S. The Technological structure and performance of developing country manufactured exports, 1985-98. Oxford development studies. 2000;28(3):337–369.
2. Balassa B. The Changing International Division of Labor in Manufactured Goods. World Bank Staff Working Paper. 1979;1(329):1–49.
3. Ross M. The oil curse: How petroleum wealth shapes the development of nations. Princeton University Press; 2012.
4. Carrera JI. La renta de la tierra: formas, fuentes y apropiación. Ediciones Imago Mundi; 2017.
