# Supplementary material for: Latent Dirichlet allocation model for world trade analysis
Source: PLoS One. 2021 Feb 4;16(2):e0245393. doi: 10.1371/journal.pone.0245393 (PMC7861422; doi:10.1371/journal.pone.0245393)
Supplement: S2 Appendix — (PDF) [file pone.0245393.s002.pdf]

S2 Appendix. Figure S2_1 shows the distribution of the top Copper producers, Chile, Peru and Zambia. While for Chile exports are almost entirely on that component [1, 2], in Peru and Zambia there is also some exports on components 9 (Coffee, bananas, other food and primary products) and 28 (Rice, cotton, textiles, gum). The resemblance of the Peruvian and Zambian exports not only on their main component, but also in these other, is an important highlight.

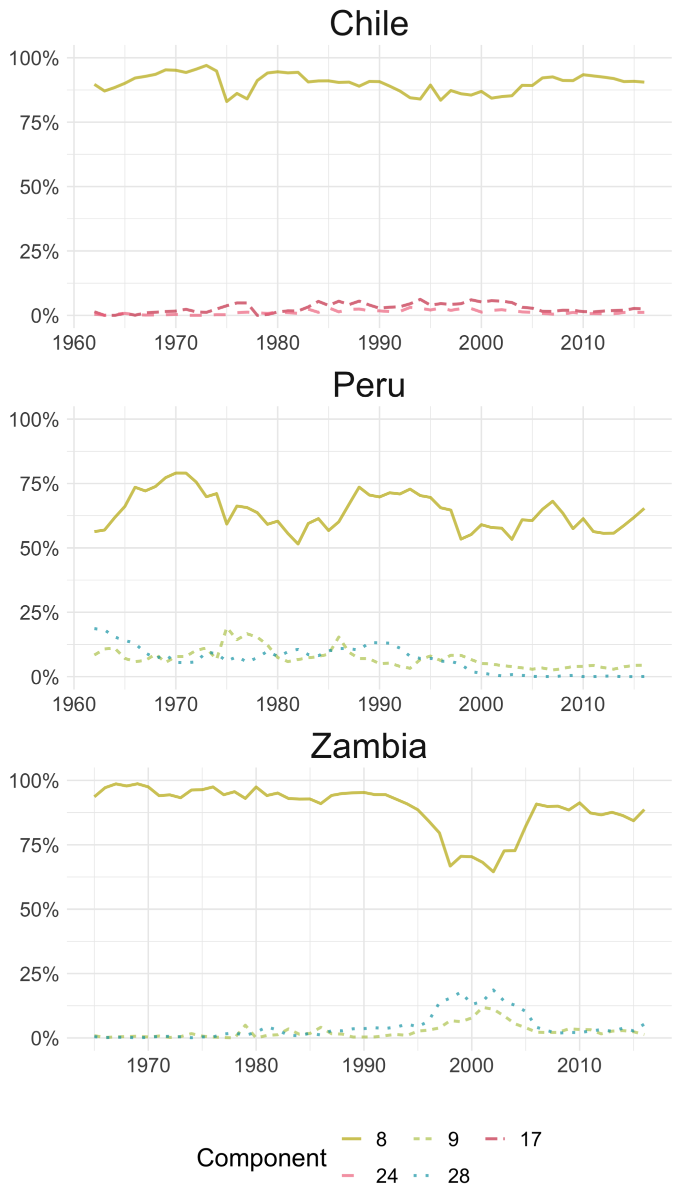

Fig S2_1. LDA outputs for Chile, Peru, Zambia. Distribution of the top three components by country. 8: Copper; 9: Coffee, bananas, other food and primary products; 17: Vehicles, parts, wood and derivatives; 24: Boats, meat, fish and dairy; 28: Rice, cotton, textiles, gum

Figure S2_2 shows the evolution of Germany, United Kingdom and the United States. In these three cases we can see a high participation of component 5 at the beginning of the series. Between 1977 and 1978 the participation of component 5 falls

abruptly, and then it continues its course until today. The component 5 includes products considered of high complexity until the break produced by the technological changes introduced by personal computers and telecommunications. In Figure S2_3 we can see how its participation evolved by decade in the ten countries that most exported in this component. We can see that for all cases the share of this component in the export decrease. In this case, the LDA model capture the obsolescence of a set of products. In the case of Germany, the results show that component 5 is replaced by the 11 (Industry of medium technology, automobiles and machinery). In the case of the United Kingdom, it is the component 30 (engines, medicines and alcoholic beverages) that takes the lead. In both cases, these components were already of significant value at the beginning of the series. For the United States, component 16 was as important as the component 5 at the beginning of the series, and after the fall of this latter, we can see an important increase in its share. Component 16 includes both heavy industry (airplanes, engines, machinery) and agricultural products (soya, corn).

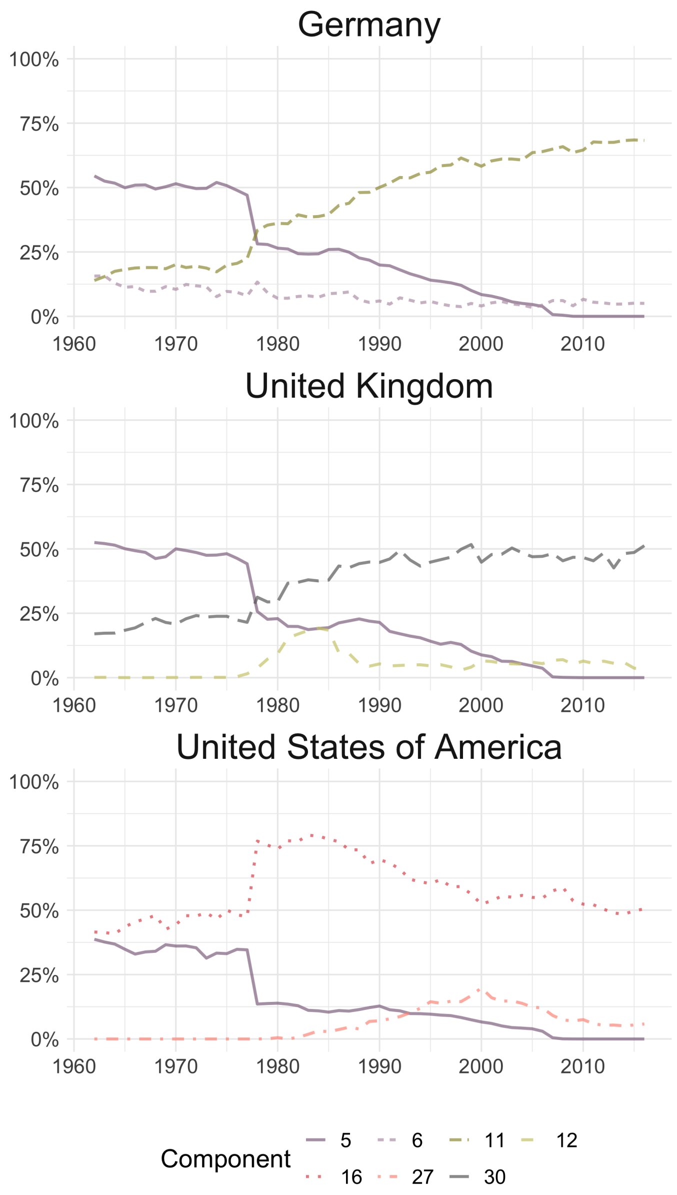

Fig S2_2. LDA outputs for Germany, United States of America and United Kingdom. Distribution of the top three components by country. 5: Non-digital electronics; 6: Vehicles, boats, machinery; 11: Cars, parts and other machinery; 12: Crude petroleum; 16: Aircraft, auto parts, soya and corn; 27: Electronic microcircuits; 30: Vehicles, parts and medicines

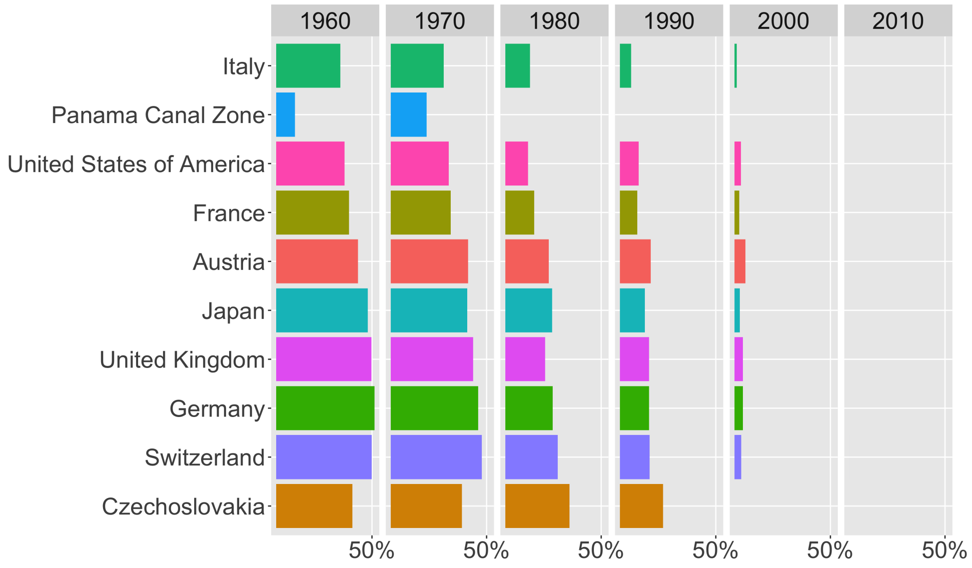

Fig S2_3. LDA outputs for Component 5: Non-digital electronics, record tapes, telephone lines, photographic paper. Distribution of the top ten countries by decade

References

1. Moran TH. Multinational corporations and the politics of dependence: Copper in Chile. Princeton University Press; 2014.
2. Mikesell RF. The world copper industry: structure and economic analysis. RFF Press; 2013.
